# The What and Where of Adding Channel Noise to the Hodgkin-Huxley Equations

**DOI:** 10.1371/journal.pcbi.1002247

**Published:** 2011-11-17

**Authors:** Joshua H. Goldwyn, Eric Shea-Brown

**Affiliations:** 1 Department of Applied Mathematics, University of Washington, Seattle, Washington, United States of America; 2 Program in Neurobiology and Behavior, University of Washington, Seattle, Washington, United States of America; Université Paris Descartes, Centre National de la Recherche Scientifique, France

## Abstract

Conductance-based equations for electrically active cells form one of the most widely studied mathematical frameworks in computational biology. This framework, as expressed through a set of differential equations by Hodgkin and Huxley, synthesizes the impact of ionic currents on a cell's voltage—and the highly nonlinear impact of that voltage back on the currents themselves—into the rapid push and pull of the action potential. Later studies confirmed that these cellular dynamics are orchestrated by individual ion channels, whose conformational changes regulate the conductance of each ionic current. Thus, kinetic equations familiar from physical chemistry are the natural setting for describing conductances; for small-to-moderate numbers of channels, these will predict fluctuations in conductances and stochasticity in the resulting action potentials. At first glance, the kinetic equations provide a far more complex (and higher-dimensional) description than the original Hodgkin-Huxley equations or their counterparts. This has prompted more than a decade of efforts to capture channel fluctuations with noise terms added to the equations of Hodgkin-Huxley type. Many of these approaches, while intuitively appealing, produce quantitative errors when compared to kinetic equations; others, as only very recently demonstrated, are both accurate and relatively simple. We review what works, what doesn't, and why, seeking to build a bridge to well-established results for the deterministic equations of Hodgkin-Huxley type as well as to more modern models of ion channel dynamics. As such, we hope that this review will speed emerging studies of how channel noise modulates electrophysiological dynamics and function. We supply user-friendly MATLAB simulation code of these stochastic versions of the Hodgkin-Huxley equations on the ModelDB website (accession number 138950) and http://www.amath.washington.edu/~etsb/tutorials.html.

## Introduction

Understanding the role of noise in cellular dynamics and function is a central challenge across computational biology. This is as true in neuroscience as in any field [1]–[3], and a universal source of noise in electrically active cells that has garnered increasing attention is the stochastic activity in ion channels [4]–[6]. This *channel noise* has been studied in a variety of neural systems including electrical stimulation of the auditory nerve by cochlear implants (e.g., [7], [8]), as well as in entorhinal cortex [9], cerebellar granule cells [10], and hippocampal CA1 pyramidal neurons [11]. Modeling studies have suggested that channel noise can influence information processing [12], spike time reliability [13], stochastic resonance [14], firing irregularity [10], [15], subthreshold dynamics [9], [10], and action potential initiation and propagation in morphologically detailed models [11], [16]. Channel noise is at work in many other systems such as the activity of cold receptor cells [17], nicotinic acetylcholine receptors [18], and calcium release by inositol 1,4,5-trisphosphate receptors [19].

Despite widespread interest in channel noise, it has remained unclear what the options are for including this noise source in a classical model of neurophysiology—the Hodgkin-Huxley (HH) equations for the action potential [20]—and related conductance-based models. The direct approach provides a gold standard for these models: each of 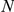 channels of a particular type transitions independently and randomly among discrete configurational states. This yields a continuous-time Markov chain with voltage-dependent transition probabilities; see [21] for a recent review. In the limit that 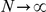 for each channel type, deterministic equations such as the classical HH equations are recovered [22]–[27]. For finite 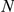, one simulates the Markov process via a Gillespie-type algorithm [16], [28]–[30].

Is there a simpler approach, where one modifies familiar models by adding a few well-placed noise terms? Beyond conceptual and computational simplicity, this would provide a direct link to powerful results on the dynamics and geometry of these differential equations [31], [32]. This line of research was initiated by Fox and Lu [22], [33], who derived candidate sets of stochastic differential equations (SDEs) using a system size expansion applied to a Markov chain version of the HH model. The past few years have seen increasing interest in this problem, spurred on by the promise, yet apparent shortcomings, of this SDE approach [10], [27], [34]–[39].

As recent work attests [27], [38], [39], accurate methods for incorporating channel noise into the HH equations are finally emerging in the form of methods both new and old. These studies demonstrate that adding noise terms to the HH equations can indeed give a compressed and accurate reproduction of the channel fluctuations. However, the placement of these terms is critical, and—as a decade of research attests—less than obvious. A key focus of our review is a unified presentation of the methods that provide the most accurate approximations to Markov chain models of channel noise. A common feature of these methods is that they introduce noise processes as *conductances* in the HH equations.

While we largely treat the original form of the HH equations—a standard reference point for neuron modeling and the focus of the prior studies we review—we emphasize that these equations are not the final word on ion channel kinetics. In fact, recent studies have pointed to alternate kinetic schemes that better capture some aspects of membrane dynamics and molecular configurations. Below, we discuss the addition of channel noise to a specific model of this type [40], [41].

## Stochastic Versions of the Hodgkin-Huxley Equations

We consider the classical equations introduced by Hodgkin and Huxley to model action potentials in the squid giant axon [20].
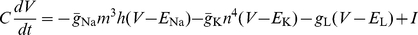
(1)

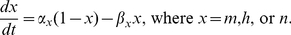
(2)Here, 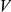 is the membrane voltage, and the gating variables 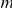, 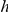, and 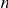 represent the fraction of open channel *subunits* of different types, aggregated across the entire cell membrane. These fractions are combined in the terms 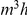 and 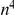 to regulate total conductances for 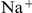 and 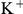 currents. The constant 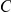 represents the capacitance of the cell membrane; 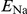, 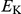, and 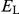 are reversal potentials; 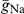 and 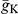 are maximal conductances; and 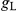 is the leak conductance.

Comprehensive introductions to this model can be found in many standard texts [23], [31], [32]. We emphasize that our discussion applies to any conductance-based model of excitable cells, including point, compartmental, or spatially extended neurons, related models of calcium release [42]. Moreover, as mentioned above it is often important to consider models with alternate schemes for channel kinetics, as we will undertake in a subsequent section for an updated model of 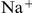 [40] and 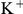 channel dynamics [41].

To model channel noise within a differential equation framework of the general form above, we seek ways of introducing fluctuations into this deterministic system. We review three approaches, which we classify as follows (and illustrate for the classical HH equations):

- **Current noise**: Replace Equation 1 with
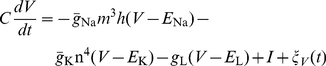
(1<*>)where 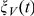 is a Gaussian white noise process.
- **Subunit noise**: Replace Equation 2 with
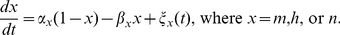
(2<*>)where the 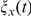 are Gaussian processes that may depend on 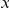 and 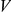.
- **Conductance noise**: Replace Equation 1 with
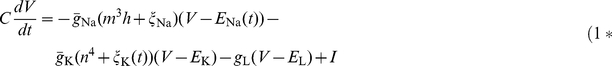
(1<**>)where the noise processes 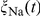 and 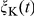 are Gaussian processes that may depend on 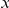 and 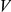.

Table 1 summarizes the differences among these models, which we now discuss in detail.

**Table 1:** Classification of channel noise models.

Noise Model	Voltage Dynamics	Subunit Dynamics	Fraction Open Na^+^ Channels	Fraction Open K^+^ Channels
None	Equation 1	Equation 2	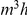	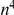
Current	Equation 1*	Equation 2	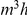	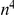
Subunit	Equation 1	Equation 2*	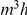	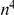
Conductance	Equation 1**	Equation 2	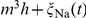	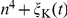

A summary of the three classes of channel noise models that we discuss in this review, and how they differ from the deterministic HH equations, which have no noise.

### Current Noise

The simplest method for incorporating noise into the classical HH equations is to add a fluctuating current term 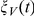 to the 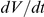 equation, as shown in Equation 1*. Here, we assume 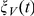 is only a function of time. Stochastic currents of this form are frequently used to drive the HH model, often in the context of the diffusion approximation for synaptic inputs [43]–[45]. In the present context, however, we emphasize that 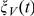 is meant to represent the combined effect of the stochastic activity of ion channels on the voltage dynamics of the cell. This approach is appealing due to its simplicity, but since channel noise is generated by the stochastic activity of ion channels in the cell membrane, it seems likely that the fluctuation term 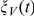 should also depend on 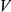 or the subunit variables. Another drawback is that, to date, there is no principled method for determining the intensity of the noise. Nonetheless, there may be cases in which current noise can be justified on empirical grounds. For instance, for a single membrane area and a constant applied current, Rowat compared the interspike interval distribution generated by a Markov chain model to the distribution generated by HH equations with current noise and found remarkably close agreement [15].

### Subunit Noise

In the HH framework, an ion channel's configuration is determined by the states of its constituent subunits, where each subunit can be either in an open or closed state [6], [23], [46]. For instance, each 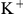 channel is composed of four 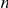-type subunits, all of which must be open in order for the channel to be permeable to 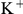 ions. Each subunit randomly transitions between its open and closed state. This suggests that the most appropriate place to add noise may be to the equations that describe the fractions of open *subunits*, as in Equation 2*. Moreover, since one typically assumes that all subunits are independent and all subunits of the same type are statistically identical, it is tempting to combine the resulting noisy fractions of open subunits to regulate conductances in the same way as one would in the deterministic HH equations; namely, by computing 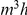 and 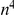.

The variables 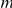, 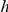, and 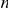 represent the aggregated fraction of open *subunits*, whereas the quantity that influences the membrane potential is the fraction of individual open *channels*. In the limit of infinitely many channels (and therefore vanishing fluctuation terms), 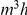 and 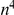 do correctly model the fraction of open channels. For a finite number of channels, however, there is no guarantee that fluctuations in the these quantities will correctly model fluctuations in the membrane-wide fractions of open channels.

To see this, note that if all channels were gated by a single subunit, then the subunit model would be appropriate—in this case, the (noisy) fraction of open subunits is identical to the (noisy) fraction of open channels. In the HH model, however, four subunits gate each channel. Combining the quantities 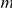, 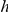, and 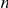 together to form the quantities 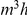 and 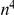 neglects the important fact that each ion channel is composed of a specific package of subunits. The states of the particular subunits within a channel, not the average state of all subunits in the cell membrane, determine whether that channel is open or closed. Thus, random transitions of individual channels among their different configurational states occur with different statistics than predicted by random transitions of the aggregated subunit variables alone [27]. This fact leads to quantitative errors produced by the subunit noise approach, as we will review below.

Subunit noise was first proposed in [22] and has been used many times; see [10], [14], [17], [19], [47]–[54], among others. By applying a system-size expansion to the states of populations of subunits, Fox and Lu arrived at a Langevin equation description of the subunit dynamics, precisely of the form of Equation 2*, where the noise terms 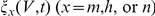 are Gaussian processes with covariance function
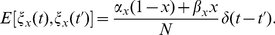
(3)Here, 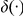 is the Dirac delta function and 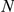 represents either the number of 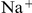 channels for the 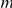 and 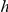 subunits or the number of 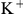 channels for the 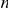 subunit. Although the authors acknowledged that the subunit noise approach has no rigorous justification and must be validated empirically, it has been widely used as an approximation to Markov chain ion channel models. However, numerical studies have revealed inaccuracies in this approximation that persist even as the number of channels increases [35], [37]. Relative to the Markov chain model, the subunit noise models produce weaker conductance and voltage fluctuations [37], [55], lower firing rates [12] (and, equivalently, longer mean interspike intervals [35]), and less variability in the occurrences and timing of spikes in response to a brief pulse of current [34], [36], and transmit information at a higher rate [12]. Furthermore, mathematical analyses of the voltage clamp statistics of the subunit noise model have proven that it does not generate stationary distributions of open channels that accurately approximate those of the Markov chain model [27], [38].

The analysis in [27] revealed similar inaccuracies in a related model proposed by [19], in which the terms 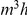 and 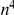 terms in Equation 1 are replaced by 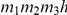 and 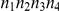, respectively, where the subscript denotes independent solutions to SDEs of the form of Equation 2*. Others have proposed simplifying Equation 3 so that the noise terms do not depend on 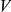, and are simply Gaussian white noise [10]. While such approaches may be justifiable on empirical grounds, in general they should not be considered as systematic approximations to Markov chain ion channel models.

### Conductance Noise

The remaining possibility is to incorporate fluctuations directly into the fractions of open channels. This seems natural, as the fraction of open channels controls ionic currents. Our intuitive understanding of the HH equations, which can be made rigorous as in [23], [25], [27], tells us that the mean fractions of open 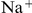 and 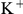 channels are given by 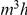 and 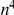. The most direct approach to adding channel noise to the HH equations, therefore, is to add zero mean stochastic processes to the deterministic values of 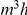 and 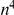. Following this idea leads to Equation 1**, which is a compact mathematical description of channel noise that preserves the original structure of the HH equations and has the biophysically desirable interpretation that channel noise induces fluctuations in the ionic conductances. We now review three channel noise models [22], [27], [38] and, with a brief set of calculations, place them in the unified framework of conductance noise.

#### Conductance noise models based on voltage clamp

Two recent studies have developed conductance noise models based on stationary statistics of channel activity in voltage clamp—called the “quasistationary” channel model in [27] and the “effective” model in [38]. Using the standard assumption that all ion channels are independent, the stationary distribution of open channels in voltage clamp is a binomial distribution parameterized by the total number of channels and the probability that any given channel is open. The probability that a channel is open depends on 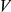, and thus a voltage clamp analysis generates a family of binomial distributions indexed by 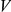, which is treated as a fixed parameter. The means of the distributions of open channels are given by familiar terms from the deterministic HH equations: 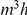 for 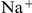 channels and 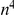 for 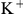 channels. If these binomial distributions are well approximated by Gaussian distributions, then the stationary distribution of open channels in voltage clamp can be accurately approximated by a family of zero mean, voltage-dependent Gaussian processes that are added to the voltage-dependent equilibrium values of 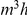 and 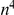.

The effective model of [38], for instance, represents the fraction of open 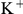 channels in voltage clamp as 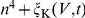 where the stochastic process 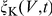 is the sum of independent Ornstein-Uhlenbeck (OU) processes (i.e., Gaussian colored noise). In other words, 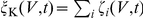, where the 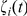 are defined by SDEs of the form:
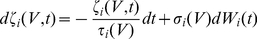
(4)with timescales 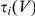 and noise amplitudes 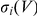 [38]. The quasistationary channel model in [27] produces equivalent Gaussian processes in voltage clamp. The difference between the two methods is that, in [27], there is a single noise process shared by all OU processes: 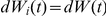 for all 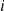 in Equation 4. While this leads to different values of 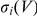, our own simulations of these models (not shown) did not reveal any systematic differences in the outputs of the two models.

To simulate such conductance noise models for a freely evolving membrane potential, one must assume Equation 4 is valid outside of voltage clamp. In practice, one numerically integrates Equation 4, where 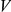 is updated in each time step according to Equation 1**. There is no assurance that this approach is valid in the context of a dynamic membrane potential. If 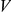 changes on longer time scales than the correlation times in the conductance fluctuations, then such an approximation may be appropriate, but an essential feature of neural dynamics is the rapid change in 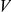 during the course of an action potential. Voltage clamp–based methods may be less reliable, therefore, for modeling the spiking activity of neurons.

These channel noise models were developed in [27] and [38] in order to approximate the original Markov chain description of channel kinetics. Their structural details—i.e., the number of 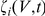 processes used to define 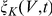 and 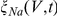 and the values of 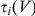 and 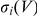 in Equation 4—were defined based on the stationary statistics of the Markov chain model. The voltage clamp approach itself, however, can be made general and model independent. The only necessary ingredients are the autocovariance functions, as a function of the voltage clamp value, for fluctuations in the conductances. Moreover, if these stationary autocovariance functions can be expressed as sums of exponential functions, then the Gaussian representation theory for multiple Markov processes ensures that they can be approximated as a linear combination of OU processes [56].

#### Conductance noise models based on Fox and Lu's system size expansion

Lacking in all of the previously discussed methods is a direct approach for modeling the dynamics of fluctuations in the fractions of open channels as the voltage 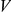 dynamically evolves. Surprisingly, the early work of Fox and Lu addressed this problem, but has apparently been overlooked ever since. Fox and Lu derived a system of SDEs in which each dynamical variable represents the fraction of ion channels in a specified configuration. This differs from their more widely used model, the subunit model discussed previously, in which the dynamical variables represent the fractions of open subunits. The resulting system of SDEs does not visibly resemble the HH equations, but with a few calculations we next show that this approach produces a conductance noise model in the form of Equation 1**.

The starting point of Fox and Lu's analysis are vectors that describe the fractions of 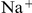 and 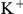 channels in each configuration as a function of time. We denote these by 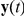 and 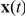. For instance, the elements of 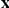 represent the fraction of 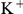 channels that have all subunits closed, three subunits closed and one open, etc. The state that will be of most interest is the conducting state, in which all subunits are open. We denote the corresponding elements of 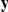 and 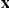 as 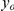 and 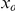, and write the current balance equation as:
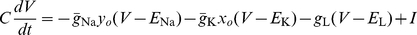
(5)The dynamics of 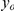 and 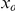 are determined by drift and diffusion matrices (see below), which Fox and Lu obtained from the original Markov chain description through a system size expansion [22], [33], [57]. We omit the details of the system size expansion, which can be found in [22], [33]. We also note that a rigorous discussion of a related method for passing from the Markov chain kinetics to a system of SDEs has been recently presented [26]. The result of Fox and Lu's expansion is a coupled system of linear SDEs of the form:
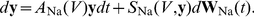
(6)

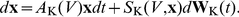
(7)The matrices 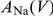 and 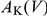 are the drift term or deterministic part of the dynamics, and are identical to the transition matrices from the master equation representation of the Markov chains for the 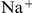 and 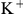 channels [22], [25], [27]. The matrices 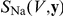 and 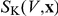 are matrix square roots of diffusion matrices; they depend on the state variable and the voltage-dependent transition rates. Stochasticity arises via the independent, standard Brownian processes 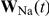 and 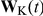.

We now demystify the connection between these equations, in which fractions of open channels are obtained from a high-dimensional system of coupled SDEs, and the standard HH equations, in which the fractions of open channels depend on the subunit variables. The key is to split the equations for 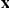 and 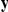 into two parts: a deterministic equation that exactly matches the gating variable equation (2), and a fluctuation equation for the noise terms. To accomplish this, we define new variables 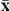 and 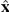, which evolve via:
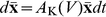
(8)

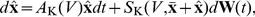
(9)with initial conditions 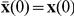 and 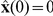. The sum 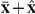 solves Equation 7, so this is an exact decomposition of 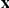 into a deterministic part 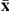 and a fluctuation part 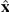. We can also apply a similar decomposition to 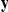. As discussed by a number of authors [23], [25], [27], solutions to the deterministic equation (Equation 8) can be generated by appropriate combinations of 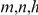, the gating variables from the deterministic HH equations: 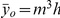 and 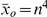. This leaves the fundamental structure of the HH equations intact. Equation 5 can be replaced by the modified HH voltage equation (Equation 1**), where the conductance noise terms 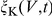 and 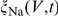 are defined to be 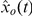 and 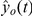, respectively.

In sum, the high-dimensional SDEs derived by Fox and Lu [22] do not modify the deterministic structure of the HH equations. Instead, as shown in Equation 9, their sole purpose is to shape the fluctuations in the fractions of open channels. An important strength of this method is that it yields a description of channel fluctuations that is equally valid outside of voltage clamp. Furthermore, as shown in [27], the stationary statistics of open channels for this method match exactly those of the Markov chain model, and it accurately replicates spiking statistics for channel numbers as small as 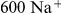 and 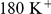 channels (membrane area of 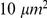).

One complication in solving these systems of SDEs is the need to determine 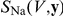 and 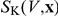 by computing matrix square roots in each time step. In order to guarantee the existence of these matrix square roots, we replace the values 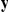 and 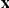 in the diffusion matrices with deterministic values obtained from the gating variables, or equivalently the solutions of Equation 8 for 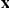 and the corresponding equation for 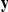.

## Comparing Stochastic Versions of the Hodgkin-Huxley Equations: Simulations

**Figure 1:**
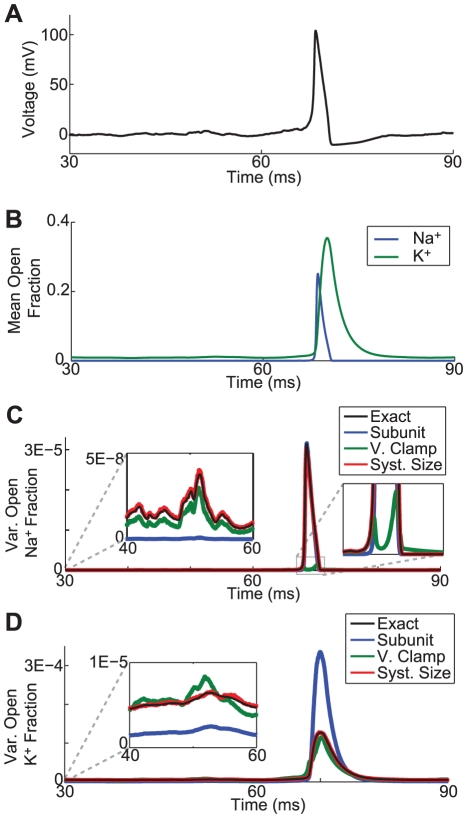
Analysis of responses of channel noise models for a fixed voltage trajectory. (A) Voltage trace obtained from the Markov chain model with no current input, 6,000 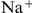 channels and 1,800 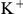 channels. Dynamics are characterized by a prolonged subthreshold period followed by a spontaneous, channel noise-induced spike at 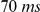. (B) Means of fraction of open 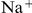 and 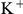 channels for the voltage trace shown in (A), as computed from Equations 10 and 11. (C) Variance in the fraction of open 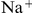 channels. (D) Variance in the fraction of open 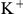 channels. Left insets in (C and D) show magnified views of the period preceding the spike. Right inset in (C) shows magnified view during the spike. For (C and D), exact variances (black) were computed from Equation 12 and Equation 13 and all other variances were estimated from 5,000 repeated simulations of the channel noise models.

**Figure 2:**
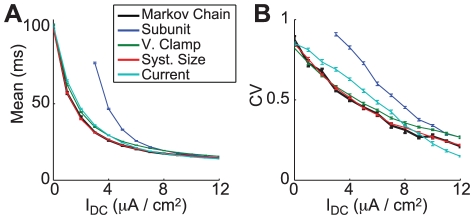
ISI statistics for DC input. (A) Mean of ISIs for a membrane area of 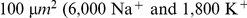 channels). (B) CV of ISIs for same membrane area as (A). 500 spikes were used to estimate the mean and variance, and error bars indicate standard error in the mean for ten repeated measurements for all models except the Markov chain model, for which only four repeated measurements were used.

How well do the simplified noise models match the “gold standard” Markov chain model of ion channel kinetics? Extensive comparisons between Markov chain and subunit noise models have been reported in prior studies [12], [27], [34], [35], [37]. Studies have also compared Markov chain models to a current noise model [15], voltage clamp conductance noise models [27], [38], and Fox and Lu's system size–derived conductance model [27], [39]. An exhaustive numerical investigation of these approaches is beyond the scope of this review, but in Figure 1 and Figure 2 we show simulation results that illustrate key differences among these approaches. All simulations use standard parameter values for the HH equations [20]. The voltage clamp conductance noise model is defined as in [38]. In all simulations, we used the Euler-Maruyama method with 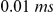 time step for solving the relevant differential equations [58] and a Gillespie-type algorithm to implement the ion channel kinetics in the Markov chain [28], [30]. To generate Gaussian pseudorandom numbers, we produced uniform pseudorandom numbers with the Mersenne Twister algorithm [59] and then transformed these using the Box-Muller method [60]. Simulation code is available upon request, and is based on the work of [38] and [27]. Both of these groups have made their code available on the ModelDB website [61], accession numbers 127992 and 128502, respectively. To complement this review, we supply user-friendly MATLAB simulation code of these stochastic versions of the HH equations on the ModelDB website (accession number 138950) and at our website http://www.amath.washington.edu/~etsb/tutorials.html.

We will first compare time-varying distributions of the fractions of open channels. Intuitively, one would expect that the number of open channels (all of which are assumed to be independent), should be binomially distributed. For a predefined voltage trajectory, this is indeed the case, as has been proven by [25]. The time-varying distributions of the fractions of open 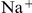 and 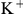 channels in a Markov chain model of ion channel kinetics approach an asymptotically stable, voltage-dependent binomial distribution with means and variances given by solutions to the deterministic subunit equations of Equation 2:
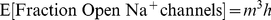
(10)

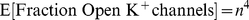
(11)

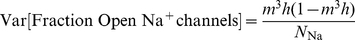
(12)

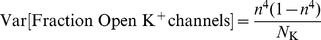
(13)We can use this result to compare channel noise models outside of voltage clamp. Figure 1A shows a single voltage trace obtained from a Markov chain model with 6,000 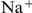 channels and 1,800 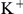 channels (membrane area 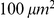) with no applied current (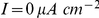). Using this sample path as an input to the channel noise models, we compare the statistics of the fractions of open channels for the different models. Figure 1B shows the mean fractions of open 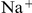 and 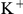 channels, as computed from Equations 10 and 11. All channel noise models produced mean values that were in close agreement with these values, so we did not plot those results.

The results for the variance of the fractions of open channels, as shown in Figure 1C and 1D, tell a different story. The variance in the fractions of open 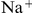 channels are computed from Equation 12 and shown in black in Figure 1C. The variance is accurately captured by Fox and Lu's conductance noise model (red), but misestimated by the subunit noise model (blue) and voltage clamp conductance noise model (green). Of particular note is the fact that the voltage clamp conductance noise model fails to track the Markov chain variance during the spike (right inset of Figure 1C). This illustrates the point, made earlier, that voltage clamp methods may not be appropriate in regimes when 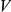 changes rapidly. The subunit noise model underestimates the variance during the subthreshold period (left inset), and overestimates the variance during the spike at 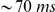 (right inset).

Figure 1D shows variances in the fraction of 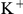 channels. Again, Fox and Lu's conductance noise model is most consistent with the equilibrium binomial distribution result. The voltage clamp model provides a reasonably close approximation, but the subunit noise model alternately undervalues the variance prior to the spike (see inset), and overvalues the variance near the time of the spike.

To illustrate the differences in the spiking activity of these models, we simulated spike trains in response to constant current inputs. In Figure 2, we show the mean and coefficients of variance (CV) of interspike intervals (ISIs) obtained from simulations of the Markov chain and SDE models. Similar simulation results have been reported in [12], [15], [27], [35]. We present results for different amounts of constant current input (*x*-axis) and a membrane areas of 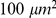 (6,000 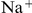 channels and 1,800 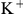 channels). The magnitude of fluctuations in the current noise model was chosen so that the mean insterspike interval of this model would match that of the Markov chain model: 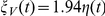 for a membrane area of 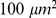, where 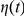 is a Gaussian white noise process with mean zero and 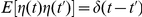.

In Figure 2A, we see that all models, with the known exception of the subunit noise model (blue), accurately reproduce the mean ISIs of the Markov chain (black), although there are slight discrepancies apparent for the current noise (cyan) and voltage clamp (green) methods. These discrepancies are even more visible when comparing the coefficient of variation of the ISIs in Figure 2B. For the conditions tested, and others reported in prior studies [27], [39], it is clear that Fox and Lu's conductance noise model (red) generates ISI statistics that are most similar to the Markov chain model.

## Beyond the Classical Hodgkin-Huxley Formulation

We have focused our discussion on the HH equations because they are a historical touchstone in the field of computational neuroscience and the subject of a large body of research on the effects and modeling of channel noise. These methods, however, can be applied to many alternative models of ion channel dynamics in excitable cells. To briefly illustrate this point, we consider an updated model of 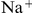 channel dynamics [40] and 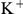 channel dynamics [41] that provide a more complete and accurate description of observed spiking activity in the squid giant axon preparation originally investigated by Hodgkin and Huxley [41], [62]. The details of this model can be found in [41].

**Figure 3:**
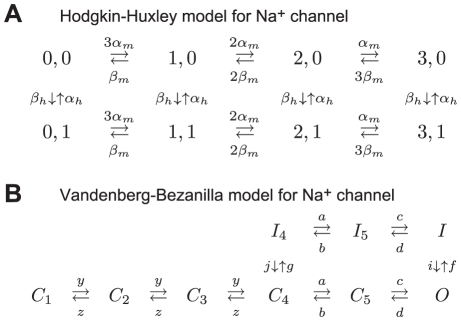
Markov chain kinetic models of the Na^+^ channel in squid giant axon. (A) Kinetic scheme for the classical HH model of the Na channel. (B) Kinetic scheme for the Vandenberg and Bezanilla model of the Na channel. Arrows are labeled with transition rates that are functions of voltage, see [20] and [41] for further details. The open states are those in the bottom right: (3,1) in (A) and 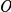 in (B).

Figure 3 illustrates the difference between the kinetic scheme for the classical HH equations (Figure 3A) and the modified model (Figure 3B). The channel is said to be open if it is in the 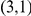 state in Figure 3A and the 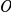 state in the modified Markov chain in Figure 3B. In contrast to the classical HH description, the modified model cannot be represented by a serial combination of identical and independent subunit particles [40]. As such, the modified model cannot be approximated with the typical subunit noise model and it provides an important test of whether conductance noise approximations can be applied to a rich set of channel configurations.

**Figure 4:**
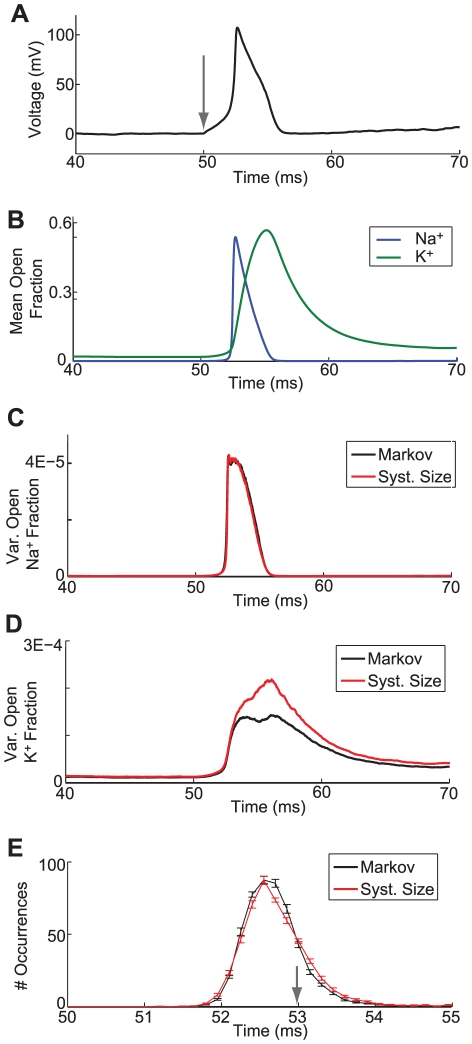
Analysis of responses of modified channel noise models to a step increase in current. (A) Voltage trace obtained from the Markov chain model with 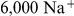 channels and 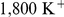 channels. Input current is increased from 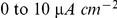 at 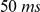, onset time of the stimulus is marked by the gray arrow. (B) Means of fraction of open 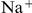 and 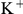 channels for the voltage trace shown in (A). (C) Variance in the fraction of open 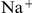 channels for the Markov chain and system size-based conductance noise models. (D) Variance in the fraction of open 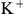 channels for the Markov chain and system size-based conductance noise models. Means and variances were estimated from 5,000 repeated simulations of the channel noise models. (E) Histogram of spike times in response to the step increase in current described above. Solid lines show the mean of ten histograms computed from 500 spike times each and error bars represent the standard error in the mean. Gray arrow indicates the spike time of a deterministic version of the model.

In Figure 4, we characterize the response of this model to a step of current that increases from 0 to 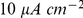 at 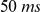. Numerical methods are similar to those described above and in [27]. A shorter time step of 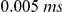 was used for these simulations. Parameter values are given in [41]. An action potential produced by the Markov chain version of this model is shown in Figure 4A with the onset time of the current step marked by the gray arrow. To test the accuracy of this SDE approximation method, we then used this voltage trace as an input to both the Markov chain and a conductance noise SDE model using Fox and Lu's system size approach. The mean fractions of open 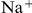 and 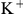 channels are shown in Figure 4B, the variances of the open 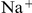 channels are shown in Figure 4C, and the variances of open 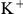 channels are shown in Figure 4D. All statistics are computed from 5,000 repeated simulations of the model using the same voltage trace (Figure 4B) as the input. For the most part, the SDE approximation accurately represents the activity of the Markov chain model, although the variance of the fraction of open 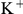 channels exceeds that of the Markov chain model following initiation of the spike.

To investigate whether these discrepancies affect the times at which spikes are generated, we study the distribution of simulated spike times for the two models in response to the same current step described above. The mean and standard error of ten histograms created from 500 spike times each and a bin size of 0.15 *ms* are shown in Figure 4E. The gray arrow marks the time at which a deterministic ODE version of this model generates a spike. The SDE model obtained using Fox and Lu's system size approximation (red line) has some bias toward producing early spikes (before 52 ms) and late spikes (after 53 *ms*), but overall the two channel noise models produce similar distributions of spike times in response to this stimulus.

In sum, it appears that the conductance noise method of Fox and Lu does accurately approximate the behavior of the Markov chain version of this modified model of channel noise in the squid giant axon, though the agreement is slightly less precise than for the classical HH framework. This points to an interesting area for future work: we anticipate that similar techniques can be applied to approximate Markov chain models of other ion channels in excitable cells, but such methods, and the details of their numerical implementation, should be compared and validated by analytical and numerical means.

## Discussion

We stand at a promising moment for the study of channel noise in conductance-based models. In recent years, due to a spate of simulation studies drawing attention to discrepancies between subunit noise models and Markov chain ion channel models [12],[27],[34],[35],[37],[38], there has been a growing sense of pessimism regarding whether SDEs could prove an effective framework for modeling the stochastic activity of populations of ion channels (e.g., [55]). However, thanks to the development of novel approximation methods [27], [38] and the rediscovery, analysis, and testing of past efforts [22], [33], new life has been breathed into the SDE approach. The validity of SDE versions of HH-type equations is now more clearly established, and the door is open for these models to generate insight into how channel effects spike timing, reliability, propagation, and other aspects of neural dynamics.

A central theme of this review is that the addition of fluctuations in conductance terms, or equivalently in the fractions of open channels, should be the preferred way for including channel noise in stochastic versions of the HH equations and related models of excitable cells. This approach, which we have termed *conductance noise*, generates models that can be directly related to the mathematical structure of underlying deterministic equations and that accurately approximate Markov chain models. In the case of the high-dimensional SDE model derived by Fox and Lu in [22], this was not obvious at first glance, and may be one reason why this aspect of their work has been overlooked. Through a brief calculation, however, we elucidated the connection between this model and the HH equations by showing how the high-dimensional SDEs can be decomposed into a deterministic part identical to the classical HH equations and a fluctuation part representing channel noise.

Although SDE models for channel noise are generally validated by making comparisons to the Markov chain model of ion channel kinetics, there is no guarantee that the Markov chain framework will remain the “gold standard.” Indeed, critiques of the Markov chain approach have been articulated (cf. [63]) and alternative mathematical models have been proposed (e.g., [64]). With this in mind, it is useful to draw a distinction between “derived models” and “empirical models.” The subunit and conductance noise models introduced by Fox and Lu [22], [33] are in the former category. They are constructed with explicit reference to the conformational states of ion channels and their subunits, as defined by a Markov chain model of ion channel kinetics. In contrast, the current noise model and the voltage clamp conductance noise models can be thought of as “empirical” since they can be constructed from observable quantities. In our simulations, for instance, we used a spontaneous firing rate to set the current noise level and the stationary statistics of open channels in the Markov channel model to define the noise processes in the voltage clamp conductance models. In principle, empirical measurements of conductance fluctuations in voltage clamp, without reference to a Markov chain model, could be used to construct channel noise models. Empirical models that can be fit to, or validated against, quantities that are readily available from electrophysiological data are an attractive direction for future research, as they may inspire new methods for incorporating channel noise in conductance-based models.

The effects of channel noise have been a subject of intense interest in computational neuroscience and related fields in computational biology. The stochastic approaches reviewed in this paper represent an important extension of the conductance-based model framework introduced by Hodgkin and Huxley [20]. Due to decades of analysis of the HH equations and an abundance of theoretical tools [65] and numerical methods (e.g., [66]) for studying SDE models, we believe that appropriate methods for adding noise processes to the HH equations and their cousins throughout electrophysiology will play an important role in the future of computational biology.
